# Bacterial signals *N*-acyl homoserine lactones induce the changes of morphology and ethanol tolerance in *Saccharomyces cerevisiae*

**DOI:** 10.1186/s13568-016-0292-y

**Published:** 2016-11-21

**Authors:** Ge Ren, Anzhou Ma, Weifeng Liu, Xuliang Zhuang, Guoqiang Zhuang

**Affiliations:** 1 Key Laboratory of Environmental Biotechnology, Research Center for Eco-Environmental Sciences, Chinese Academy of Sciences, Beijing, 100085 People’s Republic of China; 2 University of Chinese Academy of Sciences, Beijing, China; 3 State Key Laboratory of Microbial Technology, Shandong University, Jinan, 250100 People’s Republic of China

**Keywords:** *Saccharomyces cerevisiae*, Quorum sensing, AHLs, Morphological changes, Ethanol tolerance

## Abstract

The bacterial quorum sensing signals *N*-acyl homoserine lactone (AHL) signals are able to regulate a diverse array of physiological activities, such as symbiosis, virulence and biofilm formation, depending on population density. Recently, it has been discovered that the bacterial quorum sensing (QS) signal molecules can induce extensive response of higher eukaryotes including plants and mammalian cells. However, little is known about the response of fungi reacting to these bacterial signals. Here we showed that *Saccharomyces cerevisiae*, as an ancient eukaryote and widely used for alcoholic beverage and bioethanol production, exposed to short-chain 3-OC_6_-HSL and long-chain C_12_-HSL appeared obvious changes in morphology and ethanol tolerance. AHLs could increase the frequency of cells with bipolar and multipolar buds, and these changes did not present distinct differences when induced by different types (3-OC_6_-HSL and C_12_-HSL) and varied concentrations (200 nM and 2 μM) of AHLs. Further investigation by flow cytometer displayed that the cells untreated by AHLs reduced cell size (decreased FSC) and enhanced intracellular density (increased in SSC), compared with the AHLs-induced cells after incubation 6 h. In addition, the long-chain C_12_-HSL could slightly increase the ethanol tolerance of *S. cerevisiae* while the short-chain HSL obviously decreased it. Our study would be valuable to further research on the interaction between prokaryotic and eukaryotic microbes, and be reference for industrial production of bioethanol.

## Introduction

Microbes communicate with each other using chemical signal molecules, termed autoinducers (AI) or quorum sensing molecules (QSM). When the signal molecules accumulate a threshold, the communicating microbes begin to alter gene expression, and therefore behavior, in response (Waters and Bassler 2005). This process, termed quorum sensing (QS), initiates many important behaviors of bacteria including bioluminescence, sporulation, toxic factors, biofilm formation and other processes (Llamas et al. 2004; Whitehead et al. 2001). *N*-acyl homoserine lactones (AHLs) are widely used for regulating QS in gram-negative proteobacteria (Lv et al. 2013). The fungal QS systems was first revealed in fungus *Candida albicans*, regulated by farnesol as auto-inducers to control filamentation (Hornby et al. 2001). Subsequently, a lot of studies show that farnesol can result in multiple physiological effects on *Saccharomyces cerevisiae* and *C. albicans*, including biofilm formation and oxidative stress (Albuquerque and Casadevall 2012). In addition, tyrosol, phenylethanol and tryptophol are all known fungal QSMs (Chen et al. 2004; Lingappa et al. 1969). In *S. cerevisiae*, phenylethanol and tryptophol as QSMs were found to regulate morphogenesis during nitrogen starvation conditions (Chen and Fink 2006).

In recent years, it is notable that AHLs have been found to induce specific and extensive response from eukaryotes including plants and mammalian cells. Proteome analyzed shows that Medicago truncatula can detect of the two AHLs, 3-oxo-C_16:1_-HSL and 3-oxo-C_12_-HSL, resulting in significant changes in accumulation of over 150 proteins, which contribute to plants resistance, protein degradation and modification, and other metabolic procedures (Mathesius et al. 2003). To mammalian cells, AHLs are found to have a function in apoptosis. Tateda et al. (2003) found that 3OC_12_-HSL effected on macrophage inhibitory protein (MIP)-2 and monocyte chemoattractant protein (MCP)-1 expression.

In past 10 years, the research of the response of fungi to the bacterial QSMs has just begun. Most of the researches focused on the two opportunistic pathogens, *C. albicans* and *Pseudomonas aeruginosa*. It has been revealed that 3-oxo-C_12_-homoserine lactone (HSL) secreted by *P. aeruginosa* and farnesol generated by *C. albicans*, both of which contain a 12-carbon backbone, influence morphogenesis by inhibiting the *Candida* cAMP/PKA pathway (Davis-Hanna et al. 2008). Besides, there are few studies on the QS among other fungus and bacteria. Based on the fact of coevolution and coexistence between bacteria and fungus for millions of years and the similarities between their QSMs, it is reasonable that the existence of more fungus can detect and response to bacterial QS signals.

*Saccharomyces cerevisiae*, as one of the most ancient eukaryotes and with the characteristics of simple growth requirement, rapid cell division and ease of genetic manipulation, is an excellent candidate for research on the effects of bacterial QS signals on eukaryotes. Moreover, *S. cerevisiae* is one of the most important ethanol producers in industry, but when alcohols accumulate a threshold, the filamentation, growth, viability and biofilm development in yeast can be effected, as QSMs (farnesol or AHLs) do (Chauhan et al. 2013). Therefore, it is meaningful to research on the response of *S. cerevisiae* to QS signal molecules for explaining the integrity of coevolution between prokaryotes and eukaryotes, and is profound to reveal the effect of QSMs on the ethanol tolerance in yeast to environmental friendly improve the production of bioethanol.

The aim of this study was to investigate the response of the model fungus *S. cerevisiae* to bacterial signals, focusing on the influence of bacterial AHLs on growth, morphology and ethanol tolerance of *S. cerevisiae*. Two typical bacterial AHLs, 3-OC_6_-HSL and C_12_-HSL, were selected for this purpose. 3-OC_6_-HSL presents the short chain AHL with carbon chains of C_6_ with 3-oxo substitutions, while C_12_-HSL is a common long chain signal molecule with 12 carbon chains without 3-oxo substitutions. The two AHLs provided good samples for evaluating the effects of diverse bacterial signal molecules on yeast, because of their differences in carbon chain length and functional groups. Our data clearly demonstrated that bacterial QSMs indeed effected on the growth and morphology of *S. cerevisiae*, and also change its ethanol tolerance, suggesting the interaction between prokaryotic and eukaryotic microbes during coevolution. Moreover, our results could be drew some reference to increase industrial production of alcohol.

## Materials and methods

### Strains, media and culture conditions

*Saccharomyces cerevisiae* W303 was used throughout the study, which was a widely used model organism. *S. cerevisiae* was grown on Yeast-Peptone-Dextrose (YPD) medium at 30 °C. YPD medium was prepared by dissolving individual components (Yeast extract 1%, Peptone 2% and Dextrose 2%) in distilled water. Solid medium was prepared by adding 2% agar powder to YPD broth. Liquid cultures were grown with agitation at 200 rpm in a shaking incubator. *Agrobacterium tumefaciens* A136, as a AHLs biosensor, was incubated on Luria-Bertani (LB) medium with tetracycline (4.5 μg/mL) and spectinomycin (50 μg/mL) at 30 °C (Lv et al. 2012). The *S. cerevisiae* W303 (ATCC 208352) and *A. tumefaciens* A136 (ATCC 51350) strains were bought from ATCC, USA.

### Growth curve and morphological observation for *S. cerevisiae*

*Saccharomyces cerevisiae* W303 were cultured in YPD plus 3OC_6_-HSL (or C_12_-HSL) with different concentration (200 nM and 2 μM) of AHLs. The two concentrations were chosen according to previous study, in which chose concentrations of AHLs from 10 to 2 μM (Mathesius et al. 2003). We selected the two relative high points (200 nM and 2 μM) for ensuring obvious response. *S. cerevisiae* was cultured in the YPD without AHLs as negative control. Each group was cultured for 48 h and harvested cells every 3 h for growth curve drawing.

The morphological characteristics of budding yeast were observed by optical microscope (Leica DM2500, Nussloch, Germany) under the magnifying 400. The percentage of budded cells was determined by counting at least 600 cells in random sights.

### Detection of AHL degraded by *S. cerevisiae*

In this study, screening for bacterial AHLs relied on bacteriological monitor systems, which consists of a phenotypic response of *β*-galactosidase activity. *Agrobacterium tumefaciens* uses TraR (highly sensitive to most AHLs) and the autoinducer to regulate conjugal transfer of the Ti plasmid (Ravn et al. 2001). To prepare the AHLs reporter plate, 100 μL of 20 mg/mL 5-bromo-4-chloro-3-indolyl-*β*-d-galactopyranoside (X-Gal) and 20 μL of 100 mg/L isopropylthio-*β*-d-galactoside (IPTG) were smeared evenly to LB agar plates. Harvested cells at 12, 36, 48 h, and the filtered supernatant after centrifugation were spotted onto the reporter agar plates with the AHL reporter strain *A. tumefaciens* A136, and incubated overnight at 30 °C.

### The morphological identification by flow cytometer

*Saccharomyces cerevisiae* treated with 2 μM C_12_-HSL (or 3OC_6_-HSL) were incubated for 6, 9, 12, 24, 36, and 48 h at 30 °C with constant shaking (200 r/min). After incubation, the cells were harvested by centrifugation and suspended in PBS. The morphological changes were measured by BD-influx flow cytometer (BD Biosciences, USA) according to cell sizes and granularity defined in the forward light scatter (FSC) and side light scatter (SSC) plot.

### Ethanol tolerance assay

The individual colony of *S. cerevisiae* was inoculated in 10 mL YPD medium, to logarithmic phase at 30 °C with constant shaking (200 r/min). After incubation, the cells were separated from the medium by centrifuging 3000 r/min for 5 min and rinsing with sterile deionized water 2 times. Finally, the cells were resuspended in 1 mL sterile deionized water and incubated at 30 °C for 6 h in order to prepare the resting cells of *S. cerevisiae*.

After 6 h, the final resting cell density was adjusted to OD_600nm_ of 1, then tenfold gradient diluted to 10^0^, 10^−1^, 10^−2^, and 10^−3^. Four microliter of each dilution was spotted onto the YPD agar plates (containing 6% (or 8%) alcohol and 2 μM C_12_-HSL (or 3OC_6_-HSL) or without AHL) and incubated at 30 °C for 3–10 days, observing the growth of *S. cerevisiae*.

## Results

### Effect of AHLs on growth characterization of *S. cerevisiae*

**Fig. 1:**
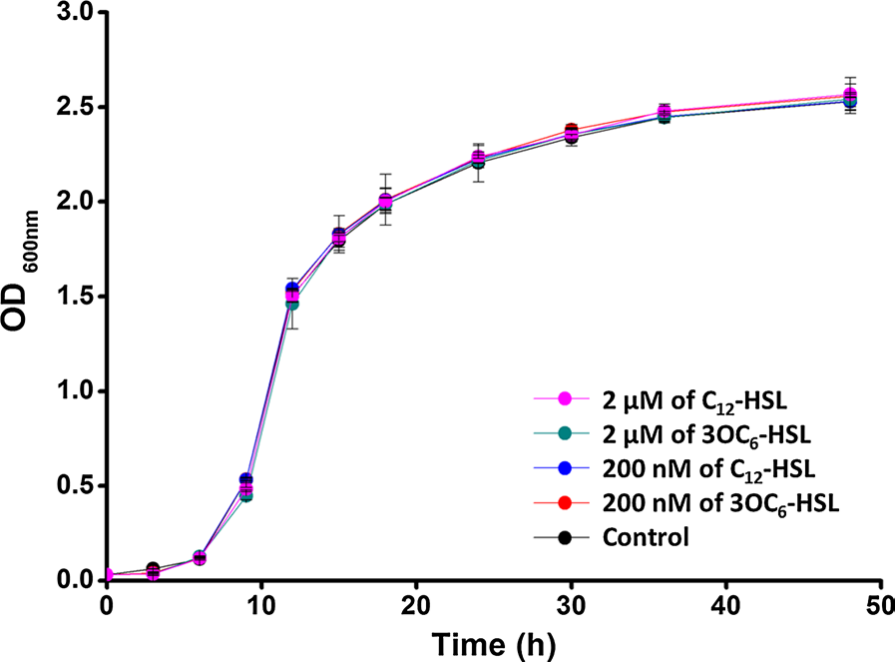
Growth curves of *S. cerevisiae* exposed to bacterial AHLs (3OC_6_-HSL and C_12_-HSL, respectively) at different concentration (200 or 2 μM). Data represented the mean ± SEM (n = 3)

To evaluate the effect of bacterial AHLs on *S. cerevisiae* growth, the cells of yeast harvested every 3 h were used to obtain the growth curve (Fig. 1). The results demonstrated that there was no obvious difference neither in each AHL-treated group nor between AHLs-treated groups and control group, which indicated that the exogenous AIs of this study had no effects on cellular growth density of *S. cerevisiae*.

### Cellular morphological observation of AHL-treated *S. cerevisiae* by optical microscope

**Fig. 2:**
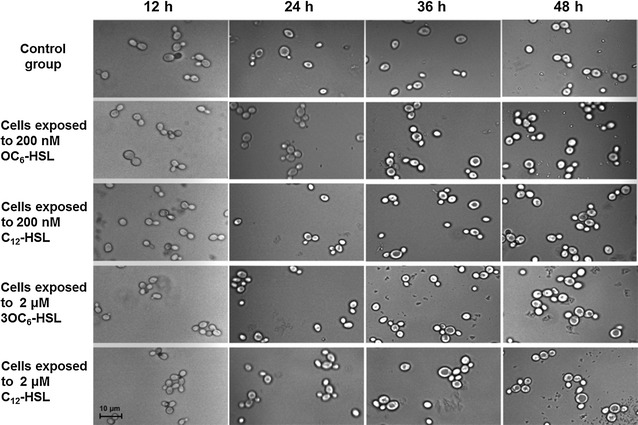
Microscopic analysis of *S. cerevisiae* cells exposed to different conditions of culture with AHLs. The conditions of culture included with two types (3OC_6_-HSL and C_12_-HSL) and two concentrations (200 and 2 μM) of AHLs. Cells without any treatment were as the control group

According to the growth curve of *S. cerevisiae* (Fig. 1), microscopic observation of *S. cerevisiae* began at 12 h when yeasts were growing at logarithmic phase. In the culture with lower concentration of AHLs (200 nM) and without AHLs treated, most cells were haploid with axial budding when exposed AHLs for 12 h, no bud cells and multipolar buddings were rare, meanwhile more diploid yeast cells with bipolar and multipolar budding presented at the group of higher concentration of AHLs-treated (Fig. 2). After AHL-treated 24 h, bipolar and multipolar budding increased obviously in AHLs-treated groups, while it rarely observed in the control group, in which cells often had one daughter budding or no buds after cultivation (Fig. 2). The influence of different chain length AHLs (3OC_6_-HSL and C_12_-HSL) on yeast morphology had not been found in this study.

**Fig. 3:**
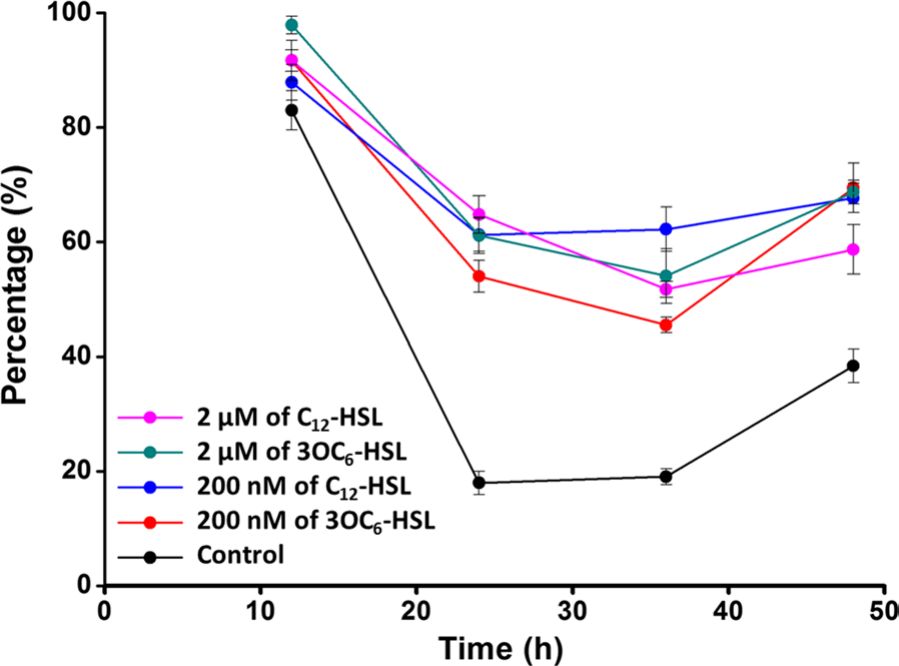
Proportion of cells with bipolar or multipolar budding after treated with different AHLs at different concentrations. *Error bars* represented the standard error of the mean for three biological replicates at each group (These values were determined by counting at least 600 cells by optical microscope)

To further verify the response of *S. cerevisiae* to AHLs, the number of axial buddings and multipolar buddings was further assessed by cell counting under a light microscope. Consist of the results of microscopic observation, the cell counting results (Fig. 3) showed that cells with axial buddings or multipolar buddings decreased sharply with the cultivation time increasing in the control group, this downtrend relieved markedly in the groups with AHLs-treated. The proportion of cells with dipolar buddings were always higher than 40% in the AHLs-treated groups, by contrast, bipolar budding accounted for only 20% of total cells in control group at the mid-cultured time.

### The morphological changes identified by flow cytometer

**Fig. 4:**
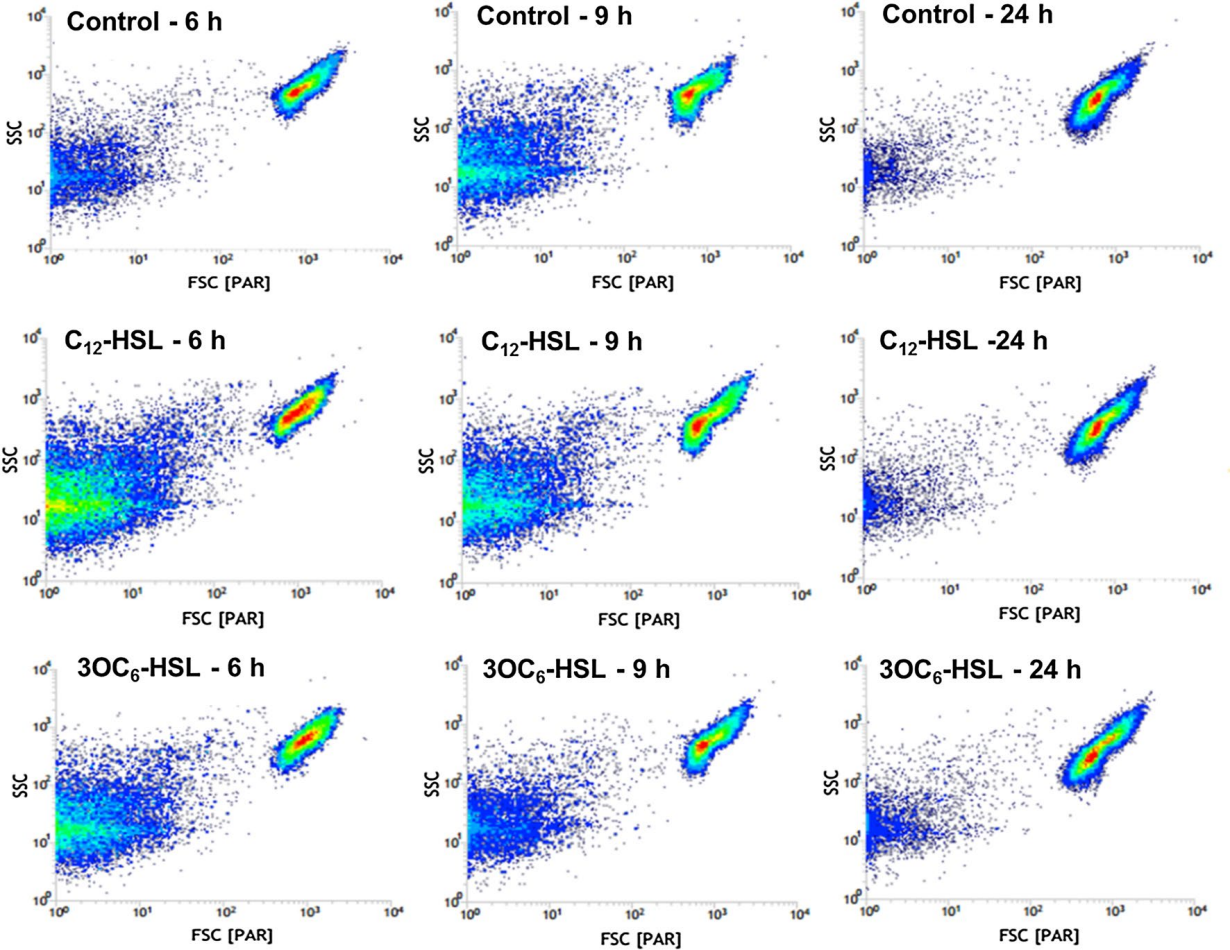
Morphological change of *S. cerevisiae* cells induced by AHLs measured by flow cytometry. FSC (*x*-*axis*) is an indicator of size and SSC (*y*-*axis*) is an indicator of granularity. *S. cerevisiae* was exposed to 2 μM C_12_-HSL and 3OC_6_-HSL, respectively. The cells without any treatment were as control

In terms of the growth curve (Fig. 1), the cells of 6, 9, and 24 h, which represented lag phase, logarithmic phase and stationary phase, respectively, were selected to perform flow cytometer. Their morphological changes induced by AHLs were determined by a flow cytometric dot plot analysis (Fig. 4) where the forward scatter (FSC) was an indicator of size and side scatter (SSC) was an indicator of granularity. After induced 6 h, the cell exposed to AHLs had a more homogeneous growth status, especially, there were high density of cells centralize on the medium level of FSC/SSC when cells were treated by 3OC_6_-HSL, however, the more cells in control showed decreased FSC and increased in SSC. When *S. cerevisiae* grown to logarithmic phase (9 h), AHLs treated samples displayed higher FSC/SSC than control samples.

### Identification of AHLs degradation by *S. cerevisiae*

**Fig. 5:**
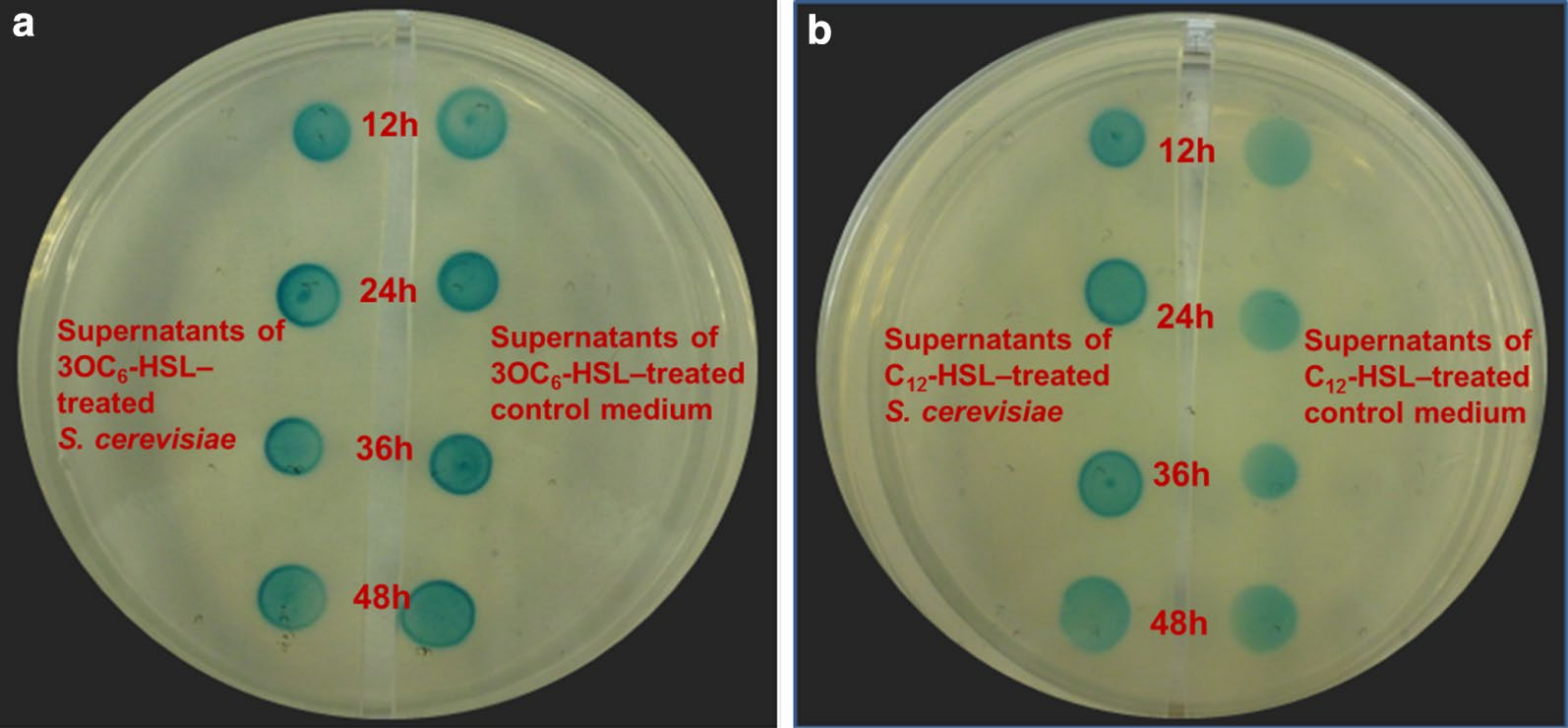
Identification of degradation of AHLs by *S. cerevisiae*. The *S. cerevisiae* strain and medium solution were incubated with 2 μM 3OC_6_-HSL (**a**) and C_12_-HSL (**b**) for 12, 24, 36, and 48 h, and then the culture supernatants were spotted onto an agar plate (containing X-Gal and IPTG). The medium solution without *S. cerevisiae* were also added 2 μM 3OC_6_-HSL and C_12_-HSL as positive control. The residual AHLs were detected by *A. tumefaciens* A136 (*blue* stain)

Although *S. cerevisiae* induced by AHLs appeared morphological changes, it needed to further confirm whether such changes were driven by the regulatory effect of AHLs or ‘carbon source’ of AHLs. The degradation test suggested that the strain did not have ability to degrade both the long-chain and short-chain AHL, when provided with 2 μM C_12_-HSL or 3OC_6_-HSL as cosubstrates in *S. cerevisiae* culture medium (Fig. 5).

### Stress resistance of *S. cerevisiae* to ethanol toxicity

**Fig. 6:**
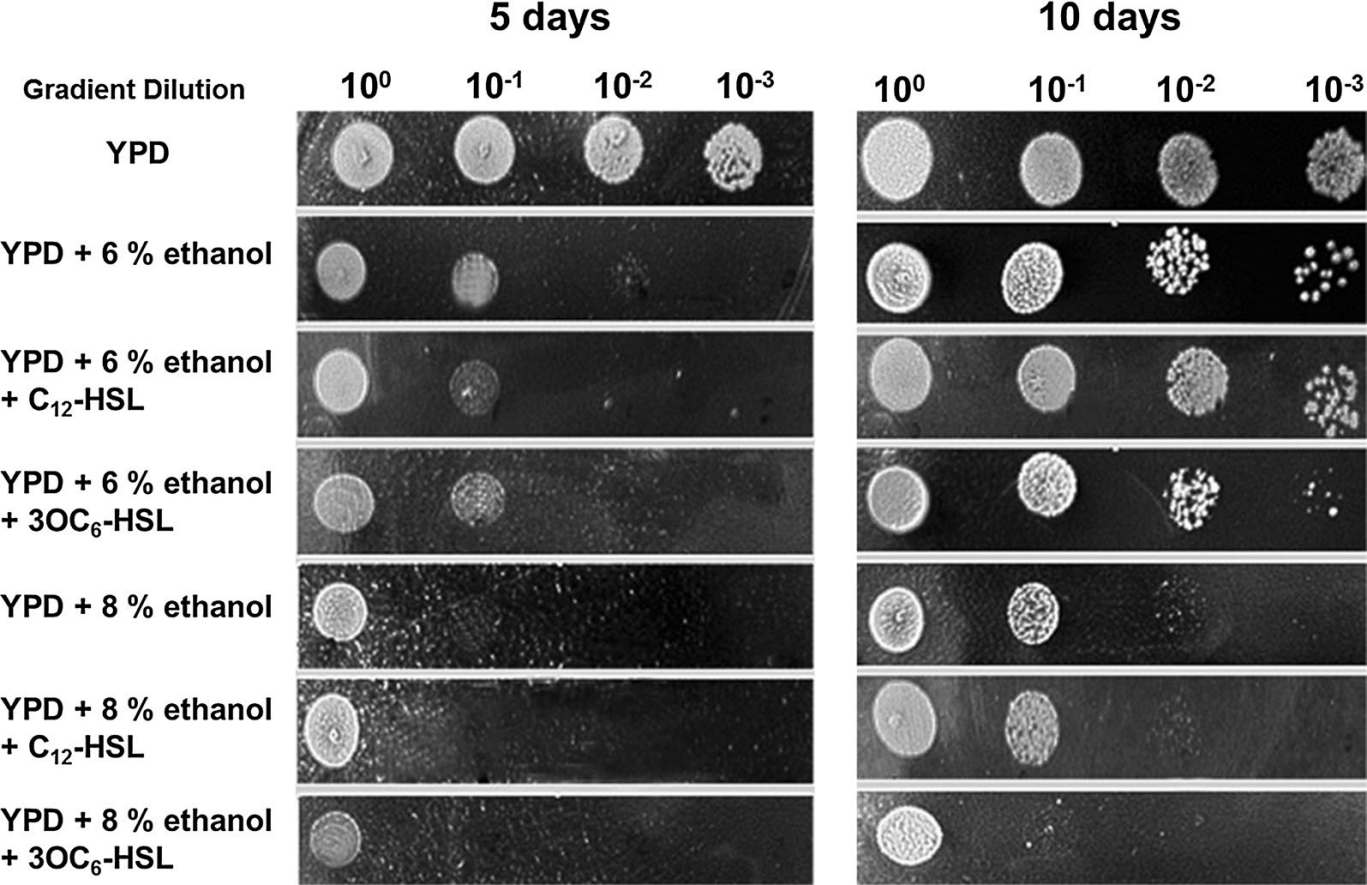
Ethanol tolerance of *S. cerevisiae* induced by AHLs. Aliquots (4 μL) of tenfold serial dilution of the *S. cerevisiae* resting cells were spotted onto YPD plates, YPD plates plus 6% (8%) ethanol, and YPD plates supplemented with 6% (8%) ethanol and 2 μM AHLs, respectively, and grown at 30 °C for 10 days. The *left*-to-*right* colonies represented the tenfold gradient dilution of 10^0^, 10^−1^, 10^−2^, and 10^−3^, respectively

**Fig. 7:**
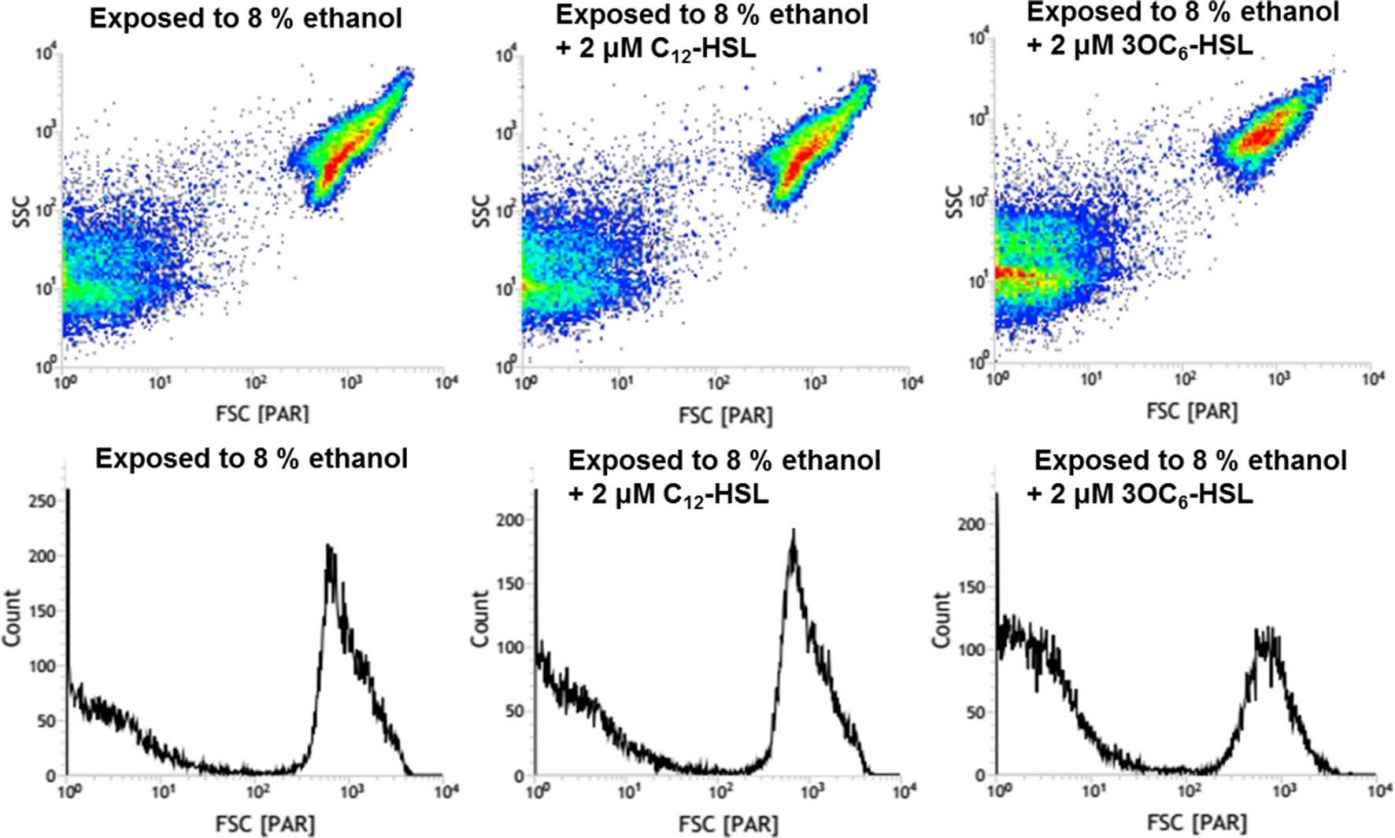
The influence of AHLs on morphology of *S. cerevisiae* stressed by 8% ethanol detected by flow cytometry. FSC (*x*-*axis*) is an indicator of size, SSC (*y*-*axis*) is an indicator of granularity, and count (*y*-*axis*) indicates cell number. 2 μM C_12_-HSL and 2 μM 3OC_6_-HSL were used in this test; the cells only exposed to 8% ethanol were as the control. The cells were incubated for 3 days before measured by flow cytometry

One of the most important characteristics of *S. cerevisiae* is the ethanol producers. Therefore, it is necessary and rewarding to research on the effects of AHLs on ethanol tolerance of *S. cerevisiae*, based on the fact that the both alcohol and AHLs can effect on *S. cerevisiae* growth. Therefore, the *S. cerevisiae* incubated on the agar plate mixed with ethanol and AHLs was used to determine the effect of AHLs on the yeast ethanol tolerance. As Fig. 6 showed, neither C_12_-HSL nor 3OC_6_-HSL influenced the ethanol tolerance of *S. cerevisiae* when incubated for 5 days. However, the long-chain C_12_-HSL slightly increased the ethanol resistance of *S. cerevisiae* after incubated for 10 days. In contrast, the short-chain 3OC_6_-HSL displayed obviously decrease in yeast ethanol tolerance (Fig. 6). The similar findings were obtained from the observation by flow cytometer (Fig. 7), that the 3OC_6_-HSL-treated cells had significantly smaller FSC value, larger SSC value and less cell number than both the long-chain AHL-treated cells and control cells.

## Discussion

*Saccharomyces cerevisiae* are similar to bacteria in using quorum-sensing to communicate with each other and coordinate certain behaviors. But the mechanism of the response from *S. cerevisiae* to bacterial QSMs has not been identified, which is significate to research on the coevolution between prokaryotes and eukaryotes, and might contribute to industrial production. This study demonstrated that bacterial QSMs, 3-OC_6_-HSL and C_12_-HSL, which cannot degrade in the culture of *S. cerevisiae* indeed regulate the morphology and ethanol tolerance of *S. cerevisiae*.

The cells with AHLs-treated had a better chance of bipolar and multipolar budding than control group (Fig. 2), although AHLs did not impact the yeast growth (Fig. 1). Therefore, we assumed that AHLs just restrained division of daughter cells from mother cells, but did not inhibit the growth of budding yeast. Similar results of previous studies showed that both fungal QS signal molecule-farnesol and bacterial QS signals-3OC_12_-HSL, which shared a 12-carbon backbone length in common, repressed hypha formation, but not growth of *C. albicans* (Davis-Hanna et al. 2008). In eukaryotic cells, it is the secondary messenger cyclic adenosine monophosphate (cAMP), which is produced in response to extracellular stimuli such as farnesol and bacterial autoinducers. The central role of the cAMP signaling pathway in the budding yeast *S. cerevisiae* is nutrient sensing and regulation of diverse biological processes including growth, metabolism, and stress resistance (Matsumoto et al. 1985). Based on these, it is reasonable that the exogenous AHLs, which act as bacterial autoinducers, cause pressure on *S. cerevisiae*, stimulating the cAMP signaling pathway. It needs to be further determined. In flow cytometry analysis, apoptotic cells always showed decreased FSC (cell shrinkage) and an increase in SSC (an increase of cell density such as chromatin condensation) (Schiller et al. 2005). In this study, after induced for 6 h, a part of cells in AHLs-untreated *S. cerevisiae* obviously showed decreased cell size and an increase in granularity. Coupled with homogeneous growth in AHLs-treated cells, it was demonstrated that bacterial AHLs could inhibit the apoptosis in *S. cerevisiae* to maintain good growth in the early stage.

In addition to the AHLs-effect on the growth and morphology of *S. cerevisiae*, the characteristic of yeast ethanol tolerance was also investigated during exposure to AHLs. The toxicity of ethanol was a major stress factor for yeast during fermentation process, which could inhibit yeast growth and viability, and to affect serious transport systems including glucose and amino acid transport (Arneborg et al. 1995; Ingram and Buttke 1984; Salmon et al. 1993). The ethanol tolerance of *S. cerevisiae* increased at the later stage of growth when treated with C_12_-HSL, by contrast, short-chain 3OC_6_-HSL-treated cells reduce the ethanol tolerance of yeast. The results of flow cytometer yielded the similar findings that the 3OC_6_-HSL-treated cells had significantly smaller FSC value, indicating the cells shrinkage. Bacterial AHLs might regulate particular genes to affect the resistance to ethanol in *S. cerevisiae*. However, ethanol tolerance is a complex phenotype and to the underlying mechanism of the resistance ethanol stress still need elucidate (Stanley et al. 2010; van Voorst et al. 2006).

The AHLs receptor proteins in *S. cerevisiae* are the most critical factor to explain both the morphological changes and the effects on ethanol tolerance during exposure to AHLs (C_12_-HSL or 3OC_6_-HSL). Theoretically, according to their hydrophobic structures and their ability to traverse bacterial cell membranes, it is possible that AHLs freely diffuse into fungal cells and bind to intracellular receptor protein. The other two possible mechanism of action for AHLs in eukaryotic cells are as modulators of intracellular biochemical reaction and as extracellular ligands for membrane-associated receptor proteins (Shiner et al. 2005). In this study, the influence of AHLs on *S. cerevisiae* particularly reflected on the morphological changes and slightly apoptosis observed by optical microscope and flow cytometer (FSC/SSC). Related researches had found amount regulatory proteins have similar effects on the *S. cerevisiae* growth, for examples, protein phosphatases which could dramatically effects on cell shape, the single yeast isozyme of protein kinase C (Pκc1p) that is capable of maintenance the cellular integrity, and HtrA-like protein Nma111P plays a crucial role in yeast apoptosis (Fahrenkrog et al. 2004; Heinisch et al. 1999; Ronne et al. 1991). Besides, the hexokinase (Augustin et al. 1965) and alcohol dehydrogenase (ADH) (Nagodawithana and Steinkraus 1976) have been shown to decrease ethanol tolerance. All of these are valuable for identification the regulation mechanisms of AHLs in *S. cerevisiae*. AHLs would probably have the similar receptor-proteins and then initiate the regulation pathways of growth or other functions in *S. cerevisiae*.

In summary, bacterial QSMs, C_12_-HSL and 3OC_6_-HSL, were capable of changing the morphology of *S. cerevisiae* and effected on the ethanol tolerance of *S. cerevisiae*, suggesting that the regulatory mechanism of bacterial QSMs to *S. cerevisiae* or other fungi might be related or similar to the pathways acted on fungal growth, morphological regulation and resistance of ethanol. It is meaningful to clarify the pathway of *S. cerevisiae* in responding to bacterial QSMs in further study, for deeply understanding the interaction between prokaryotic and eukaryotic microbes and increasing ethanol tolerance of yeast in industrial production.

## Abbreviations

AHL: *N*-acyl homoserine lactone
QS: quorum sensing
AI: autoinducer
QSM: quorum sensing molecules
HSL: homoserine lactone
YPD: Yeast-Peptone-Dextrose
LB: Luria-Bertani
FSC: forward light scatter
SSC: side light scatter
cAMP: cyclic adenosine monophosphate
